# Development of a Solid and Flexible Matching Medium for Microwave Medical Diagnostic Systems

**DOI:** 10.3390/diagnostics11030550

**Published:** 2021-03-19

**Authors:** Amin Moradpour, Olympia Karadima, Ivan Alic, Mykolas Ragulskis, Ferry Kienberger, Panagiotis Kosmas

**Affiliations:** 1 Keysight Technology Labs, 4020 Linz, Austria; mykolas.ragulskis@keysight.com (M.R.); ferry_kienberger@keysight.com (F.K.); 2 Faculty of Natural and Mathematical Sciences, King’s College London, Strand, London WC2R 2LS, UK; olympia.karadima@kcl.ac.uk (O.K.); panagiotis.kosmas@kcl.ac.uk (P.K.); 3 Biophysics Institute, Johannes Kepler University, Gruberstr. 40, 4020 Linz, Austria; ivan.alic@jku.at

**Keywords:** microwave imaging system, microwave measurement, dielectric properties measurement, matching medium, antenna measurement, microwave system calibration

## Abstract

This paper reports the development of a new composite material as a matching medium for medical microwave diagnostic systems, where maximizing the microwave energy that penetrates the interrogated tissue is critical for improving the quality of the diagnostic images. The proposed material has several advantages over what is commonly used in microwave diagnostic systems: it is semi-flexible and rigid, and it can maximize microwave energy coupling by matching the tissue’s dielectric constant without introducing high loss. The developed matching medium is a mirocomposite of barium titanate filler in polydimethylsiloxane (PDMS) in different weight-based mixing ratios. Dielectric properties of the material are measured using a Keysight open-ended coaxial slim probe from 0.5 to 10 GHz. To avoid systematic errors, a full dielectric properties calibration is performed before measurements of sample materials. Furthermore, the repeatability of the measurements and the homogeneity of the sample of interest are considered. Finally, to evaluate the proposed matching medium, its impact on a printed monopole antenna is studied. We demonstrate that the permittivity of the investigated mixtures can be increased in a controlled manner to reach values that have been previously shown to be optimal for medical microwave imaging (MWI) such as stroke and breast cancer diagnostic applications. As a result, the material is a good candidate for supporting antenna arrays designed for portable MWI scanners in applications such as stroke detection.

## 1. Introduction

The utilization of non-ionizing electromagnetic (EM) waves at microwave frequencies for medical imaging is a technology that has been widely investigated in the last years. Microwave imaging (MWI) systems are portable, cost-effective as well as safe due to the usage of non-ionizing radiation. Therefore they can be used as an alternative or complementary modality for existing medical imaging systems such as magnetic resonance imaging (MRI) and computed tomography (CT) [1,2,3] with potential applications in breast cancer and brain stroke detection [4,5]. MWI systems typically use a vector network analyzer (VNA) to generate and record the microwave signals and an array of antennas to transmit and receive the EM energy interacting with the interrogated region in the body. These signals can then be translated to diagnostic information using various signal processing and imaging techniques. A critical aspect of many MWI systems is the use of an interface medium known as a matching medium that has a complex relative permittivity of $\varepsilon_{m}$ with the real part $\varepsilon^{\prime }$ and the imaginary part $\varepsilon^{\prime \prime }$.

The impact of matching media on the diagnostic capabilities of medical MWI systems has been demonstrated in various studies [6,7,8,9,10,11,12,13]. For example, the benefits of using a lossy dielectric as matching medium in microwave tomographic breast imaging have been argued in [8]. These include a reduction of the model error and hence of image artifacts, which lead to detection enhancement. The paper proposed glycerine combined in various proportions with saline as a coupling and immersion medium and studied mixtures with relative dielectric constant values in the range from 30 to 77 and conductivity values from 1.35 to 1.78 S/m. The optimization study in [9] used a one-dimensional plane-wave model of skin-covered fatty breast tissue to find the best values of relative permittivity for breast imaging systems. Using four different numerical breast models, Angiulli et al. [10] investigated the influence of lossless matching media on the quality of 3D image reconstructions and concluded that the optimal dielectric constant values are in the range 5–25.

For stroke detection applications, the influence of a matching medium for a microwave stroke diagnosis was investigated in [11]. Multi-layer planar tissue models of healthy and hemorrhagic stroke brain were used to study the effect of various matching media on the reflected and transmitted power. The paper reported that a matching medium with low relative permittivity results in a better contrast between healthy and hemorrhagic brain. In contrast, the analysis in [12] reported that the matching medium of a MWI system should have a relative permittivity between 10 and 40 to optimize the penetration of microwave signals inside the head.

Beyond stroke and breast cancer detection, the impact of the matching medium’s conductivity on a biomedical microwave tomography system designed for limb imaging was studied in [13]. A mixture of water and various concentrations of table salt was used as the matching medium and the effect of different mixtures on the quality of reconstructed images was investigated. It was demonstrated that low-loss coupling media lead to reconstructions of low quality due to an increased modeling error. On the other hand, using a high-loss immersion medium increases the noise in the system, and consequently results in poor quality of the reconstructed images. It was concluded that 2.5–4.5 g of table salt per liter of deionized water provide the best reconstruction quality for this limb imaging system.

Considering the dielectric mismatch between air and human tissue, a matching medium is typically used to couple the EM energy to the body. This helps to minimize the reflections and improve the transmission to the body-air interface. Immersing the antenna array inside a lossy matching medium has additional advantages such as broadening the antenna bandwidth and reducing unwanted reflections from the imaging tank [14]. On the other hand, the medium’s additional losses will attenuate the already weak signal response from a target of interest, such as tumor or stroke. Consequently, a wide dynamic range and a high signal to noise ratio are required to record the target reflections above the noise level. Depending on the imaging method (tomographic or radar-based), the matching medium’s dielectric contrast can resemble the properties of skin or normal body tissues [15,16,17].

The open-ended coaxial probe is the most common technique to measure the dielectric properties of a candidate matching medium. The coaxial probe method employs a truncated coaxial transmission line which propagates the EM field along its axis. The reflections happen where the EM field faces an impedance mismatch between the probe and the sample material. The reflections are then measured by the VNA and the material’s dielectric properties are calculated [18].

This paper focuses on the development and characterization of a new material as a matching medium for MWI systems. The proposed material is a micro-composite of barium titanate (BaTiO${}_{3}$) and polydimethylsiloxane (PDMS) mixed in weight-based ratios. To measure the dielectric properties of the resulting materials with the different mixing ratios, the open-ended coaxial probe method is used. Furthermore, repeatability of our measurements as well as homogeneity of the developed material is addressed. Finally, to evaluate the developed matching medium’s impact, we compare reflection coefficient measurements of a printed monopole antenna with and without the matching medium around it.

## 2. Materials and Methods

### 2.1. Permittivity Measurement Set-Up

**Figure 1:**
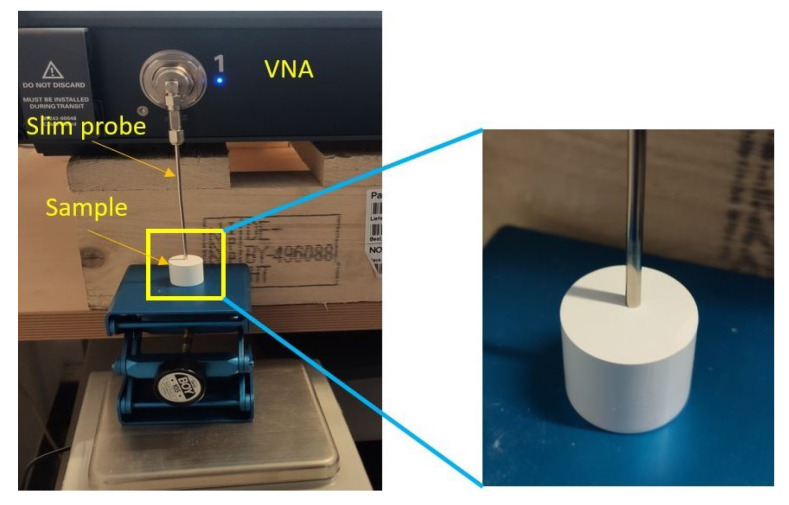
Dielectric properties measurement set-up. The Keysight slim probe is placed on the sample after a full one-port calibration.

The dielectric properties measurements of the materials were performed with the Keysight N5230C PNA-L microwave network analyzer. Keysight’s materials measurement suite N1500A [19] is used as the software for data processing, calibration and calculation of the dielectric properties. Furthermore, the open-ended coaxial slim probe from Keysight 85070E dielectric probe kit is used as a measurement probe. Figure 1 shows the measurement set-up. To increase the stability of the measurement system and to eliminate the cable effects, we directly connect the probe to the VNA. Before each set of measurements on the samples, the whole system is calibrated with the standard references of air, conductive textile as short and distilled water at room temperature. The calibration theory and mathematics of the system is outlined in the “Section 2.2”. The repeatability of the measurements will be described in the “Section 3”. To increase the accuracy of the measurements, the calibration is refreshed on every repeated measurement, and air is used as the refreshing standard. The frequency span was from 500 MHz to 10 GHz with 401 and 201 frequency points, using both linear and logarithmic sweeps. The power level used was −10 dBm and the intermediate frequency bandwidth was selected to be 50 Hz.

### 2.2. System Calibration

**Figure 2:**
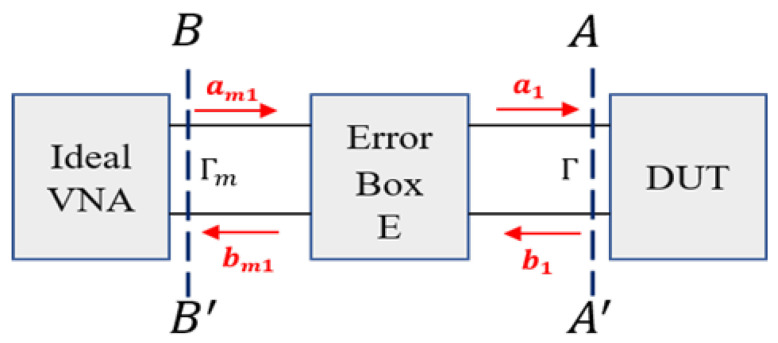
One-port error box, which includes both unwanted effects by the probes and the instrument’s systematic errors.

To calculate the dielectric properties of the material accurately, we need to obtain the reflection coefficient at the aperture of the probe (denoted as A-A${}^{\prime }$ plane in Figure 2) from VNA measurements of the reflection coefficient on the B-B${}^{\prime }$ plane. Both instrument systematic errors and unwanted probe effects are modeled in the error box depicted in Figure 2 and are corrected by applying one port calibration technique [20], which determines the error parameters in the error box. Once the parameters are calculated, the reflection coefficient can be corrected.

In Figure 2, Γ represents the true value of the reflection coefficient while $\Gamma_{m}$ is the measured reflection coefficient measured by the VNA. The error coefficients can be written as [20]:

(1) $$\left[\begin{array}{c} b_{m1}\\ a_{1} \end{array}\right]=\left[\begin{array}{cc} e_{11} & e_{12}\\ e_{21} & e_{22} \end{array}\right]\left[\begin{array}{c} a_{m1}\\ b_{1} \end{array}\right]$$

We can calculate the true reflection coefficient at the A-A${}^{\prime }$ plane from (1) as:

(2) $$\Gamma =\frac{\Gamma_{m}-e_{11}}{e_{22}\Gamma_{m}-\Delta }$$

where Δ suggests $e_{11}e_{22}-e_{12}e_{21}$. The error coefficients $e_{11}$, $e_{22}$ and Δ are calculated from:

(3) $$\left[\begin{array}{ccc} 1 & \Gamma_{1}\Gamma_{m_{1}} & -\Gamma_{1}\\ 1 & \Gamma_{2}\Gamma_{m_{2}} & -\Gamma_{2}\\ 1 & \Gamma_{3}\Gamma_{m_{3}} & -\Gamma_{3} \end{array}\right]\left[\begin{array}{c} e_{11}\\ e_{22}\\ \Delta \end{array}\right]=\left[\begin{array}{c} \Gamma_{m_{1}}\\ \Gamma_{m_{2}}\\ \Gamma_{m_{3}} \end{array}\right]$$

During the calibration, $\Gamma_{1}$, $\Gamma_{2}$, $\Gamma_{3}$ are three reflection coefficients from known calibration standards. In this work, we use water, air and conductive textile as three calibration standards.

### 2.3. Matching Medium Preparation

#### 2.3.1. Material Properties

BaTiO${}_{3}$ is a dielectric ceramic material that possesses piezoelectric, ferroelectric, dielectric and thermometric properties. It has a wide range of industrial applications such as multilayer ceramic capacitors, thermistors, microwave absorbers, transducers and electro-optic devices. The properties of BaTiO${}_{3}$ are dependent on the particle size, purity and crystalline phase resulting from the preparation method [21,22]. Its dielectric properties strongly depend on the grain size, and a very dense ceramic of BaTiO${}_{3}$ can have relative permittivity values as high as 7000 at 1 kHz [23]. It is reported in [24] that pure BaTiO${}_{3}$ shows values of relative permittivity around 105 in the X-band, but mixing BaTiO${}_{3}$ with other materials like PANI drastically reduces the values of complex permittivity and loss tangent of the mixture (adding only 5% of PANI reduces the relative permittivity to 26). Such studies show that BaTiO${}_{3}$ permittivity values can drop heavily when mixed with other materials, and this is what we also observed in our experiments in Section 3.1. BaTiO${}_{3}$ is also bio-compatible and thus suitable for direct contact with the human body in imaging and therapy systems. Consequently, BaTiO${}_{3}$ nanoparticles have been used for drug delivery and as label-free imaging probes [25].

Polydimethylsiloxane (PDMS) is a widely used silicon-based organic polymer. It is optically clear, inert, non-toxic, and non-flammable as well as biocompatible and gas-permeable. Unmodified PDMS is hydrophobic, however surface treatment with oxygen plasma can make the surface temporarily hydrophilic [26]. Aqueous solvents will not infiltrate and swell the material, but most organic solvents (with exception of some alcohols) will diffuse into the material and cause it to swell. High oxygen and carbon dioxide permeability allow cell respiration, therefore PDMS is suitable for microfluidic cell cultures applications [27]. PDMS applications range from soft lithography, microfluidics chips and microelectromechanical systems to medical devices and cosmetics [27,28].

#### 2.3.2. PDMS-BaTiO${}_{3}$ Micro-Composite Preparation

To prepare the matching medium samples, we mixed BaTiO${}_{3}$ powder with particle size less than 3 μm with PDMS in various mixing ratios. Both materials were purchased from Sigma-Aldrich. To take into account the duration of the degassing step and ensure the mixture is completely degassed before it solidifies, we chose a PDMS with long setting time. Previous research has stated that particle size around 1 micron is a good choice for high permittivity [23].

**Figure 3:**
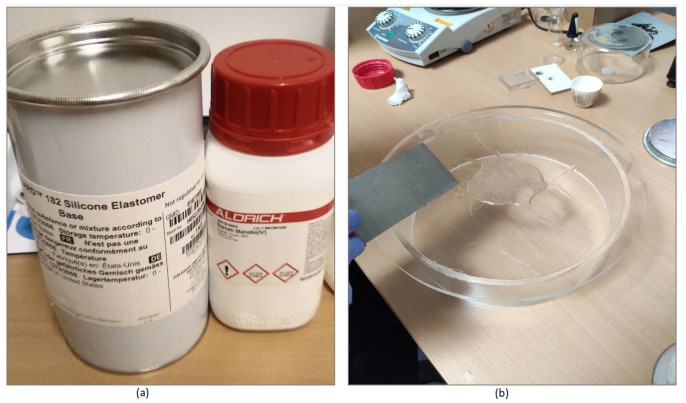
Photos of the experimental procedure: (**a**) the materials used to make the composite, and (**b**) snapshot of the mixing process.

We now describe the steps involved in the process of making the composite. First, we measure the weight of the BaTiO${}_{3}$ powder and the PDMS to achieve the desired mass ratio. Then, we mix the PDMS with its curing agent, using a 10:1 ratio, according to the manufacturer instructions. This ratio can be adjusted to achieve different material stiffness, noting that hardness and flexibility are mostly influenced by the amount of the BaTiO${}_{3}$ powder. Then, PDMS is placed on a flat surface and we add part of the powder on top. Using an elastic steel or a plastic spatula, we press the powder into the silicone and mix it thoroughly. The procedure of pressing and mixing the material is repeated until there are no clumps. Powder is gradually added until the entire amount of powder is used. The mixing step is critical for removing the clumps of powder and to obtain a homogeneous material. Next, for the degassing step, we use a low pressure vacuum with a pressure of 0.1 bar. If the pressure is lowered too rapidly, the mixture will start to foam and can leak out of the mold. Using slow setting PDMS is crucial as degassing can take up to 4 h at 0.1 bar. The mixture is being checked for bubbles after the container is repressurized by scraping off the surface and observing if any gas is still trapped under it. The degassing step is necessary as many gas bubbles can make the sample unusable. Finally, after the mixture is set, we observe that it is stiffer than pure PDMS. However, it can be cut and machined easily to create molds. Figure 3 shows a snapshot of the mixing process as well as the used materials.

## 3. Results

### 3.1. Prepared Composites

Five different mixture ratios of the proposed matching media were prepared and the dielectric properties measurements were performed following the methods described in Section 2. The prepared materials under test (MUT) are provided in Table 1.

**Figure 4:**
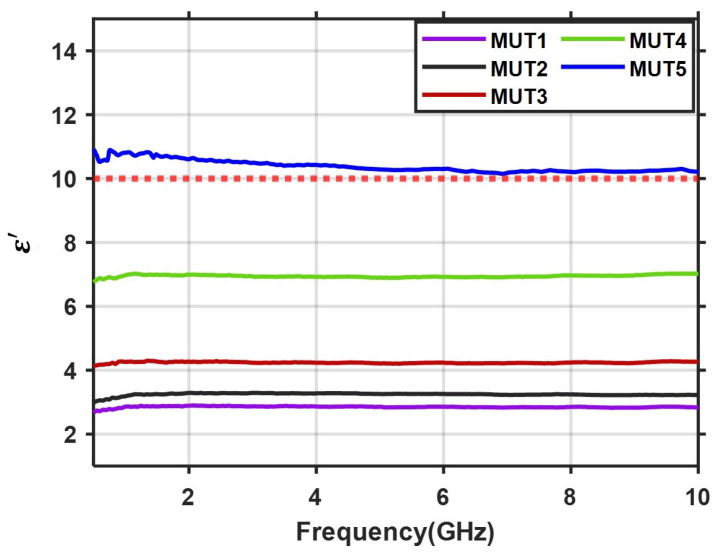
Measured dielectric constant values for the five MUTs using the process described in Section 2. The red dotted line represents the minimum recommended value for stroke detection, as calculated in [12].

The measurement results for different samples are depicted in Figure 4 which shows a nonlinear relation between the amount of BaTiO${}_{3}$ and the relative permittivity. By adding more BaTiO${}_{3}$ to the mixed material, we can increase the $\varepsilon^{{}^{\prime }}$ values. However, increasing the BaTiO${}_{3}$ causes less flexibility and makes the degassing step more difficult during the sample preparation. From the depicted results in Figure 4, it is possible to find an empirical formula at a frequency point of interest, however this is valid only for BaTiO${}_{3}$ ratios from 10% to 70%. From our preparation of samples we recommend that the maximum percentage of BaTiO${}_{3}$ in the mixture should not exceed 80%, Otherwise, it becomes very difficult to mix and degas the sample.

Figure 4 suggests that all five mixtures shown in Table 1 can be good candidates as matching and immersion materials for microwave medical imaging, depending on the selected approach (radar or tomographic) and the body region of interest. For example, in [6], a numerical study has been conducted to assess the performance of a radar-based imaging technique in the presence of matching media that are identical to oil ($\varepsilon_{r}=3$), fatty tissue ($\varepsilon_{r}=9$) and skin ($\varepsilon_{r}=36$). The evaluations show that the best quality images are reconstructed when the matching liquid with $\varepsilon_{r}=3$ is selected. Comparing this result with our developed materials, we believe that all of our proposed MUT1, MUT2 and MUT3 are proper candidates for such diagnostic systems.

In another study [10], four different numerical breast models are used to investigate the effect of lossless matching medium on the quality of 3D image reconstruction in breast tumor diagnosis. The results show that the optimal value of relative permittivity for each of the four phantom models is different ($\varepsilon_{f}=5$, 10, 15 and 22 respectively). Based on these results, we suggest that by properly selecting the mixing ratio of BaTiO${}_{3}$ and PDMS, it is feasible to develop the matching medium with relative permittivity values of 5 and 10, which can be appropriate candidates for such diagnostic systems. The mechanical properties of the composite (semi-flexible and easy to mold) suggest that it can be attractive for stroke detection and monitoring, for which a permittivity value in the range of 10–40 has been proposed as optimal in [12]. Therefore, we selected MUT5, which satisfies this requirement, for further experiments to test the material’s homogeneity as well as the repeatability of the measurements.

### 3.2. Repeatability Evaluation of Permittivity Measurements

**Figure 5:**
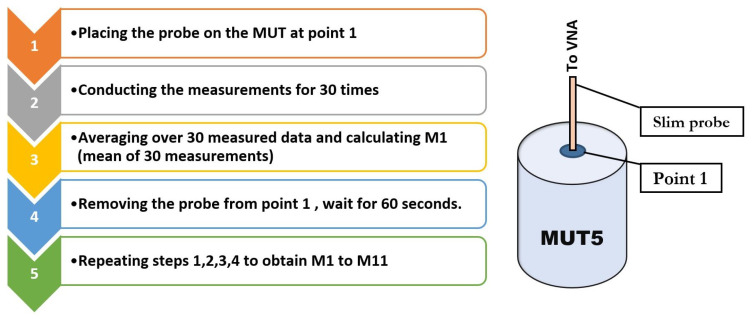
Repeatability measurement steps for MUT5 at one single point.

**Figure 6:**
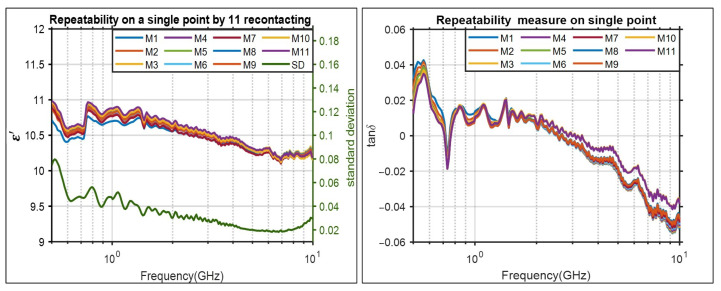
Repeatability evaluation of the permittivity measurements. Recontacting is performed 11 times and 30 measurements are conducted each time. Each of the 11 curves is the mean of the 30 measurements. The standard deviation of the 11 measurements is calculated and depicted as well.

The experimental process for assessing the repeatability of our measurements is summarized in Figure 5. In the first step, the slim probe is placed on the MUT5 at point 1 and the measurement is repeated 30 times. In the second step, the probe is removed from the sample, and after 60 s it is placed on the point 1 again (recontacting). In the next step, the probe’s pressure on the sample is controlled and adjusted to be the same as the previous contact. Once the pressure is adjusted, the measurements are conducted for additional 30 times. Finally, the total 330 measurements (11 times recontacting by 30 times measurement) are collected and presented in Figure 6.

As it is shown in Figure 6, the standard deviation of the 11 measurements is very small (below 0.08 in all frequencies) which suggests very good repeatability. The measured values for the loss tangent are below 1 in the frequency band of interest, which indicates that the material can be approximated as lossless for frequencies up to 10.0 GHz. Very small values of loss tangent translates to less attenuation in the medium, and consequently better transmission of power to the tissue, which highly affects the quality of reconstructed image in MWI diagnostic systems. We note that the negative values are due to the instrument inaccuracy since the coaxial probe technique is not suitable for loss tangent values below 0.05. We note that an alternative method to capture the loss tangent accurately for very small values is by utilizing various microwave resonators at different specific frequencies of interest, but this is beyond the scope of this work.

### 3.3. Material Homogeneity Evaluation

**Figure 7:**
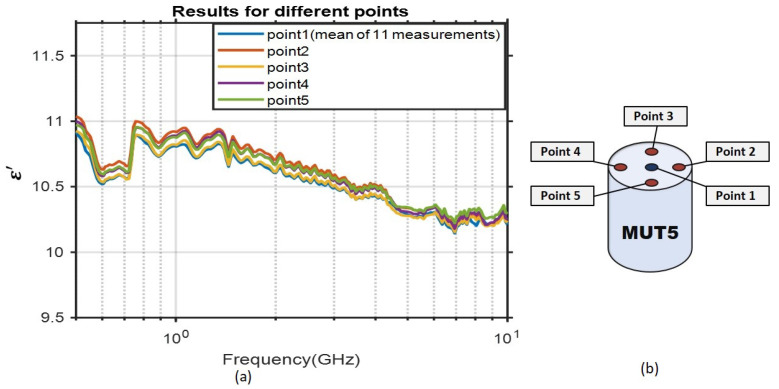
Homogeneity evaluation of MUT5. (**a**): The permittivity result of five different points. (**b**): The positions of the five different measurement points.

The material properties must be consistent through all the sample to ensure a homogeneous matching medium. To assess the homogeneity of the sample, five different points were selected on the MUT5, and measurements were performed at each point. The probe’s pressure was adjusted to be equal on all the selected points. The results and the positions of selected points are depicted in Figure 7.

We observe that the results are almost identical. The small differences between measurements is mainly due to experimental errors from the probe pressure on different points.

### 3.4. Functionality Assessment

**Figure 8:**
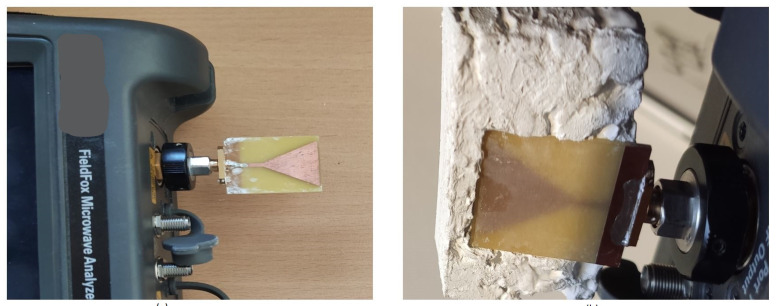
(**a**) The antenna is not covered by the matching medium. (**b**) The antenna is covered by the matching medium. The antenna is directly connected to the VNA in both cases.

To evaluate the effect of the matching medium on an antenna used in microwave imaging, we measured the reflection of a printed monopole antenna backed by our composite. The antenna has been used previously in a microwave tomography system, where it was immersed in a lossy glycerol-water mixture [14]. To remove unwanted radiation from cables, the antenna was directly connected to the VNA, and two sets of measurements were performed for comparison. In the first case, the antenna was left in open space and the reflection coefficient S${}_{11}$ was measured using Keysight FieldFox VNA. In the second case, the antenna was covered by the matching medium and the S${}_{11}$ was measured again. Figure 8 shows the setup for both cases.

**Figure 9:**
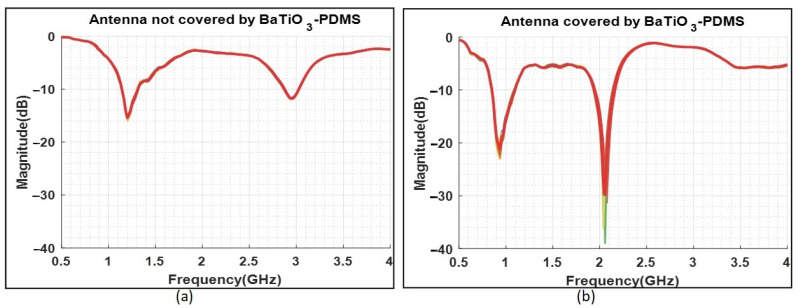
(**a**) S${}_{11}$ measurements while the antenna is not covered by the matching medium. (**b**) S${}_{11}$ measurements when the antenna is covered by the matching medium. In both cases, the measurements are performed 10 times and the red bold line is the mean of all measurements.

The results are depicted in Figure 9. For each case, measurements were conducted ten times on two different days (five measurements per day). The bold red line is the average of all measured data which overlaps with individual measurements.

As shown in Figure 9, the reflection is decreased by 6 dB in the first resonance frequency when using the matching medium in the system. The effect of the coupling medium is more significant in the second resonance frequency, where we observe an almost 18 dB reduction in S${}_{11}$ magnitude. More importantly, the matching medium increases the antenna bandwidth from 0.82 to 1.1 GHz, which is of great interest for brain imaging. A similar bandwidth of 0.8 to 1.2 GHz has been achieved for a brick antenna proposed recently for microwave brain imaging [29]. Moreover, the use of the proposed matching medium allows operation in the 1.9–2.2 GHz band, which can be important for improving resolution in microwave breast tomography [30].

## 4. Discussion

In this paper, we have developed a new coupling medium for MWI systems in medical diagnosis. As previously pointed out, the presence of a proper matching medium in a microwave-based medical imaging system reduces the reflection signals and increases the transmitted power to the human tissue which causes higher quality of the reconstructed image and thus the accuracy of the diagnostic system. Many papers have studied the influence of coupling medium on MWI systems in different applications such as brain stroke and breast tumor detection. To sum up, findings from different studies [6,7,8,9,10,11,12,13] indicate that the choice of optimal value of relative permittivity is not fully explored and depends on many factors.

We believe that the proposed matching medium and its method of preparation has many important advantages. Firstly, we are able to obtain various dielectric constant values from 2 to 11 by a proper mixing ratio of BaTiO${}_{3}$ and PDMS in a controlled manner. This enables us to prepare permittivity values of interest just by changing the mixing ratio in an easy and precise manner. Secondly, the process of preparing this coupling medium is affordable and does not need specialized equipment. Thirdly, the loss tangent values of the proposed composites are very low (below 1) for all its different compositions, which allows us to achieve an increase in the dielectric constant values without introducing losses in the medium. This cannot be accomplished with many conventional mixtures used in the MWI literature, such as glycerol-water solutions. Our proposed lossless medium allows the EM signals to be better transmitted and this can enhance the accuracy of diagnostic systems. On the other hand, if we need the medium to be lossy as in some MWT systems, salt can provide a means to do this easily, and this possibility will be investigated in future work. Moreover, while most matching media proposed in literature are liquid, our matching medium is semi-solid in its final stage of preparation and can be machined to support conformal arrays in brain or breast imaging applications.

Our future work will focus on studying this material in more detail. In particular, continuing from our experiments which demonstrated that it is feasible to immerse a MWI antenna into the proposed medium and enhance its reflection coefficient, we aim to examine the performance of an antenna array constructed with this material and compare imaging results with those of a prototype immersed in glycerol-water mixtures [14]. Applications of interest include breast and stroke detection using either radar or tomographic approaches.

## Figures and Tables

**Table 1:** Five different materials under tests (MUTs) were investigated. Each has different BaTiO${}_{3}$ content from 10% to 70%.

	BaTiO${}_{3}$(%)	PDMS(%)
MUT1	10	90
MUT2	20	80
MUT3	30	70
MUT4	50	50
MUT5	70	30

## Data Availability

Not applicable.
